# Rumination in bipolar disorder: evidence for an unquiet mind

**DOI:** 10.1186/2045-5380-2-2

**Published:** 2012-01-23

**Authors:** Sharmin Ghaznavi, Thilo Deckersbach

**Affiliations:** 1 Massachusetts General Hospital, Boston, MA, USA

**Keywords:** bipolar disorder, rumination, executive functioning, emotion regulation

## Abstract

Depression in bipolar disorder has long been thought to be a state characterized by mental inactivity. However, recent research demonstrates that patients with bipolar disorder engage in rumination, a form of self-focused repetitive cognitive activity, in depressed as well as in manic states. While rumination has long been associated with depressed states in major depressive disorder, the finding that patients with bipolar disorder ruminate in manic states is unique to bipolar disorder and challenges explanations put forward for why people ruminate. We review the research on rumination in bipolar disorder and propose that rumination in bipolar disorder, in both manic and depressed states, reflects executive dysfunction. We also review the neurobiology of bipolar disorder and recent neuroimaging studies of rumination, which is consistent with our hypothesis that the tendency to ruminate reflects executive dysfunction in bipolar disorder. Finally, we relate the neurobiology of rumination to the neurobiology of emotion regulation, which is disrupted in bipolar disorder.

## Introduction

Bipolar disorder is characterized by episodes of mania or hypomania, with or without one or more episode(s) of depression. By recent estimates, it affects between 1 and 2.5% of the general population in the United States [1]. Although mania/hypomania is the distinguishing feature of bipolar disorder, recurrent depressive episodes constitute the most frequent and functionally debilitating, unresolved aspect of the illness for individuals with bipolar disorder [2,3]. For example, over their lifetime, patients with bipolar disorder experience many more depressive episodes than manic or hypomanic episodes and spend longer amounts of time depressed than manic or hypomanic [4,5]. They are also more likely to consult a physician or psychiatrist for depression rather than for mania [6]. Likewise, depressive episodes are associated with comparatively greater occupational and psychosocial disruption then manic/hypomanic episodes. This includes impairments at work [6,7] as well as disruptions of patients' family and social life [6]. Unfortunately, despite the advancements in pharmacotherapy and the emergence of adjunctive psychosocial treatments, the diagnosis and management of bipolar depression remain significant challenges [8,9].

## Melancholic depression in bipolar disorder

One of the earliest observations about bipolar depression is that it is more likely to be a *melancholic *depression. According to DSM-IV, the *melancholic *subtype is a depressed state characterized by anhedonia, excessive weight loss, psychomotor agitation or retardation, insomnia, worsening of symptoms in the morning, early morning awakening and excessive guilt. Although operational definitions of *melancholia *have varied over the years and across diagnostic systems (DSM III [10], DSMIII-R [11], Research Diagnostic Criteria [12], the World Health Organization Depression Scale [13], the Newcastle Scale - Versions I and II [13,14], Hamilton Depression Rating Scale [15]; see [16] for a review), the one consistent feature across the various definitions has been that of psychomotor retardation [16], described as a slowed or decreased rate of movement and/or speech.

In one of the early studies of the phenomenology of depressed states, Dunner and colleagues [17] found that in the midst of a depressive phase, inpatients with bipolar I disorder showed significantly less attention to personal appearance and exhibited greater psychomotor slowing than inpatients with major depressive disorder (MDD). Similarly, in a study looking at melancholic depression in patients who met criteria for melancholic depression based on three different definitions, including DSM-III, the rate of bipolar disorder was significantly higher than unipolar depression, regardless of the definition of melancholia employed [18]. They also found that melancholia was most clearly distinguished by psychomotor disturbance. Likewise, in a study by Mitchell and colleagues [19] that compared bipolar I disorder patients with patients with MDD, patients with bipolar disorder were found to be more likely to have psychomotor retardation and atypical features (such as hypersomnia and leaden paralysis) than depressed patients with MDD.

The generally held belief that patients with bipolar disorder (in particular bipolar I disorder) are more likely to experience a melancholic depression characterized by psychomotor retardation than patients with MDD, as well as the research showing that melancholic depression is more common in bipolar I disorder, has led to the notion, among clinicians, that there is a corresponding mental slowing as well [20,21]. However, there have been no studies to suggest that patients with bipolar disorder are less mentally active. The notion that there is mental slowing in bipolar depression may also be, in part, a contrast to the large body of evidence that points to an active mind in major depressive disorder, in the form of rumination.

## Rumination in major depressive disorder

Major depressive disorder (MDD), sometimes referred to as unipolar depression, is characterized by one or more episodes of depression, without any episodes of mania or hypomania. Thus, it is nosologically distinguished from bipolar disorder by the absence of hypomanic or manic episodes. One feature of MDD which has received considerable attention is the tendency to ruminate [22], that is responding to negative affect or depressed mood by focusing on self and symptoms of distress, without actively engaging in active problem solving [23].

Rumination represents a behavioral and attentional style of responding to negative affect or depressed mood. Thus, rumination is distinguished from such problems as indecisiveness, as well as a set of recurrent thoughts about death and suicide. Similarly, rumination is distinguished from dysfunctional attitudes, which are a set of general beliefs about the self, world and future which are negatively biased, as opposed to a means of responding to a negative affective state. In fact, dysfunctional attitudes are thought to be present in individuals at risk for depression when they are euthymic (that is, not in a negative affective state).

By the same token, rumination is also distinguished from negative automatic thoughts, which refers to a set of thoughts with distorted negative content. As Nolen-Hoeksema originally pointed out, patient's ruminative thoughts may often in fact be realistic rather than distorted (for example, "I can't complete my work on time.") These distinct features of rumination were demonstrated in early studies of rumination in which participants were asked to state their responses to prompts in a rumination induction paradigm developed by Nolen-Hoeksema and colleagues [24]. While it might be argued that yet another difference between rumination and automatic thoughts is that there seems to be a volitional component to a ruminative response style, whereas negative automatic thoughts seem less volitional, such a characterization is oversimplified. As discussed later, the cognitive underpinnings of rumination suggest that while a ruminative response style may start off as seemingly volitional, its perpetuation may not be volitional.

The tendency to ruminate is highly correlated to depressed mood (see [22] for a review). To date, much of the research on rumination has been carried out on individuals with dysphoria and patients with MDD [22]. Early studies on rumination found that rumination can maintain or even worsen depressed mood [25,26]. Additionally, the tendency to ruminate predicts the likelihood to go on to develop a first major depressive episode following a stressor [23], as well as a worse prognosis in patients with major depression. The tendency to ruminate was found to be predictive of higher levels of depressed mood at discharge and follow-up after hospitalization in a group of inpatients with MDD [27]. Studies on the phenomenology of rumination reveal that it is a repetitive and persistent phenomenon that is difficult to stop and maladaptive [28-30]. To date, there are no studies examining whether rumination is more prevalent in certain types of depression (for example, excited depression or melancholic depression); however, such research might illuminate differences in the phenomenology of mood states in different types of depression.

Rumination is related to a negative self-concept in individuals with major depression. In fact, in the course of their research on rumination, Nolen-Hoeksema and Morrow [31] developed a rumination induction task, which consists of self-focused but neutral statements, (for example, "think about the sensations in your body"). When healthy normal controls are asked to engage in the task, they do not show any changes in mood. However, when depressed individuals are asked to perform the same task, they report maintenance or even worsening of depressed mood [31]. Presumably, self-focus promoting statements activate negative self-schemata, which trigger corresponding negative thoughts associated with maintenance or worsening of mood in depressed patients, thereby creating a vicious cycle of increased self-focus, negative thoughts and depressed mood.

Studies of negative thoughts and beliefs as assessed by two widely used and well validated measures, the Automatic Thoughts Questionnaire (ATQ)[32] and Dysfunctional Attitudes Scale (DAS) [33], show that negative automatic thoughts and dysfunctional attitudes, often related to the self, are elevated in depressed states (ATQ: [32,34-37]; DAS: [33,37-40]). This does not appear to be the case when patients are euthymic (ATQ: [34,37,38]; DAS: [37-40]). A few studies have also found that dysfunctional attitudes interact with negative life events, or stress, to predict depressive symptoms [41,42]. Thus, latent negative self-concepts, when activated, as in rumination, may trigger depressed mood and relapse into depression.

## Negative self-concept in bipolar disorder

There is an emerging literature documenting persistent negative self-concepts (schemata) in individuals with bipolar disorder as well. In one study, using the ATQ and DAS, Hollon *et al*. [37] found that regardless of nosology (MDD, bipolar disorder, substance induced depression), among patients who were currently depressed, scores on both the ATQ and DAS were higher than in other clinical and control groups. Additionally, the ATQ covaried directly with levels of depression as measured by the Beck Depression Inventory (BDI). In a study directly comparing scores on the DAS between patients with bipolar disorder and patients with MDD, Jones and colleagues [43] found that, when current levels of depression were taken into account, there was no difference on scores on the DAS between the two groups. More recently Scott and Pope [44] also found that patients with MDD and bipolar disorder did not differ significantly on overall scores on the DAS. The finding that scores on the DAS and ATQ do not differ between patients with MDD and bipolar disorder suggests that self-concept in depressed states in both disorders is equally negative.

Consistent with this, Reilly-Harrington *et al*. [45] found patients with bipolar disorder and MDD performed similarly on a Self-Referent Information Processing Task [46] that consisted of a battery of four tasks which tap into self-attributes. Both groups performed the tasks in a manner consistent with a negative self-concept.

Additionally, research on self-esteem suggests that patients with bipolar disorder and major depression demonstrate similar low levels of self-esteem. In their study comparing patients with bipolar disorder and MDD on the DAS, Scott and Pope [44] also used the Rosenberg Self-Esteem Questionnaire (SEQ; [47]). The SEQ is a measure of trait self-esteem consisting of five negative and five positive statements (corresponding to negative and positive self-esteem respectively), which participants rate on a 1 to 4 scale. They found that patients with bipolar disorder and MDD did not differ significantly on overall scores on the SEQ. Similarly, in the Jones and colleagues study [43], when current levels of depression were taken into account; there was no difference on scores on the SEQ between depressed patients with bipolar disorder and depressed patients with MDD, and both patient groups had significantly lower scores on the SEQ than control participants.

Unique to bipolar disorder, scores on the DAS are elevated in patients with bipolar disorder during the manic phase as well as the depressed phase. Goldberg and colleagues [48] compared scores on the DAS in patients with bipolar disorder who were manic, patients with MDD who were depressed, and controls. They found that while patients with MDD, who were currently depressed, had the highest scores on the DAS, patients with bipolar disorder who were manic, had significantly higher scores on the DAS than healthy controls.

As in MDD, scores on the ATQ and DAS do not appear elevated when patients with bipolar disorder are in remission. For example, in the study by Hollon and colleagues [37], they found that scores on the ATQ and DAS were not elevated compared to controls, in patients with bipolar disorder who were in remission. Likewise, in a study by Lex and colleagues [49] investigating cognitive styles in patients with bipolar disorder in full remission, there were no significant differences on scores on the DAS and ATQ between patients with bipolar disorder who were in remission and normal controls.

Finally, similar to patients with MDD, negative self-concept or schemata seem to predict relapse into mania and depression in patients with bipolar disorder. In the study by Reilly-Harrington and colleagues [45], scores on the DAS interacted with the number of negative life events to predict increases in depressive, as well as manic, symptoms. Depressed patients with bipolar disorder, who possessed greater dysfunctional attitudes and experienced more negative life events, showed worsening in their mood symptoms, both depressive and manic symptoms.

## Rumination in bipolar disorder

While the bulk of the research on rumination has been conducted in individuals with major depression, given the research showing negative self-concept/schemata in bipolar disorder and the role of rumination or self-focus in sustaining depressed mood one might expect that depressed patients with bipolar disorder engage in ruminative thinking, similar to patients with MDD. Indeed, this is what Johnson *et al*. [50] found in a recent study looking at the tendency to ruminate in individuals with bipolar disorder, MDD and normal controls. They used the Response Styles Questionnaire (RSQ; [51]), which is a measure of the tendency to engage in ruminative responses when feeling depressed, and found that both individuals with MDD and bipolar disorder endorsed heightened rumination in response to negative affect, compared to normal controls.

Consistent with this, van der Gucht *et al*. [52] found that the tendency to ruminate was highly correlated with depressive symptoms, (as assessed by the Hamilton Depression Rating Scale; HDRS), in patients with bipolar disorder. In a recent study, in our group with patients with bipolar disorder, using the RSQ, we also found that patients with bipolar disorder, who are depressed, show a greater tendency to ruminate, when compared to levels previously reported in normal controls [53].

Given the repetitive and persistent nature of ruminative thinking, its presence in depressed states in patients with bipolar disorder is contrary to the widely held belief that depression in bipolar disorder is characterized by mental slowing, decreased mental activity, or a relative dearth of thought. Early studies of rumination in unipolar depressed states, in which participants were asked to state their thoughts in response to the prompts on the rumination task developed by Nolen-Hoeksema and colleagues, demonstrate that individuals who ruminate are mentally quite active, with numerous thoughts, albeit with a negative bias [24]. Patients with bipolar disorder in depressed states ruminate and, as such, are mentally quite active, suggesting an unquiet mind in depressed states in bipolar disorder as well.

Notably, melancholia and rumination are not necessarily contrasting phenomena. As reviewed above, various definitions of melancholia exist, but generally these definitions, including the current DSM-IV definition, encompass primarily physical symptoms (for example, psychomotor disturbance). Thus, cognitive states in melancholia are not well characterized. It is possible, given the greater depressive symptom load in melancholic depression and the correlation between rumination and depressive symptom load, that rumination is prevalent among individuals afflicted with melancholia. Thus, rumination and melancholia are not necessarily contrasting phenomena, and, in fact, it is likely that rumination is a key feature of melancholia, not previously described.

What appears to be unique to bipolar disorder is that studies of rumination in bipolar disorder reveal that not only do individuals with bipolar disorder ruminate in response to negative affect in depressed states, they ruminate in response to positive affect as well. In the study by Johnson *et al*. [50], individuals with bipolar disorder endorsed ruminating in response to positive affect as well as negative affect, albeit with a more positive focus. Using the Response to Positive Affect Questionnaire [54] which assesses tendencies in responses to positive affect, Johnson and colleagues found that individuals with bipolar disorder, who were hypomanic, endorsed a tendency to focus on positive affective experiences and positive self-qualities. Thus, patients with bipolar disorder engage in a positive ruminative style in response to positive affect in a hypomanic state, as well as a negative ruminative style in response to negative affect, in a depressed state. Notably, positive rumination is conceptually different from grandiosity, which is defined as an inflated sense of self-esteem or believing that one has special powers, spiritual connections or religious relationships. Ruminative thoughts of a positive nature may often be realistic (for example, "I did a good job on a project at work last week", which describes a positive affective experience that is reality based). Additionally, positive rumination is distinguished from flight of ideas by the fact that the content of positive ruminations is organized around positive affective experiences and qualities, and not random associations. Studies which record the content of positive ruminations, as Nolen-Hoeksema and colleagues have done with negative rumination [24], are needed to provide empirical support for these distinctions.

Surprisingly, the tendency to engage in rumination to negative affect may also be associated with hypomania. In studies of college sample students [55,56], scores on the RSQ are found to be positively correlated with hypomanic traits (as assessed by the Eckblad and Chapman's Hypomanic Personality Scale; HPS [57]). Individuals who have a tendency to focus on their symptoms of distress and negative self-qualities also possess more hypomanic traits. Notably, in another study, van der Gucht *et al*. [52] did not find a correlation between rumination (as assessed by the RSQ) and manic traits (as assessed by the Bech-Rafaelson mania scale [58]) in patients with bipolar disorder.

## Psychological accounts of why patients with bipolar disorder ruminate

Given the role of rumination in persistence of negative mood, the question arises, why do depressed patients ruminate? One account that has been put forward to explain rumination in unipolar depression by Papageorgiou and Wells [28] is that rumination is a coping strategy. Papageorgiou and Wells found that patients identified advantages as well as disadvantages to rumination, suggesting that they engage in rumination in part because of a perceived benefit from rumination. The advantages cited by patients included a sense of improved understanding of their depression and the causes of their depression, and a sense of control over their symptoms and problem solving.

This account is likely applicable to patients with bipolar disorder who are depressed. They may engage in rumination for reasons similar to their MDD counterparts. However, such an account does not explain why rumination has been associated with hypomania in patients with bipolar disorder as well. One possibility that has been put forward to account for this finding [55] invokes the age old idea of the manic defense to depression. On this account, the hypomania/mania is a coping strategy just like rumination, so the correlation between hypomania and rumination reflects that they are both coping strategies used by patients with bipolar disorder who are struggling with depression. Support for this account comes from a recent study by Goldberg and colleagues [48] showing that patients with bipolar disorder in a manic phase endorse negative core beliefs, suggesting that the mania may be a means of coping with the negative core beliefs. Another account is based on research that patients with bipolar disorder generally have higher expectations [59] and are goal attainment focused [60], but when depressed, have greater difficulty realizing those expectations and goals. The account proposes that this difficulty leads to a failure to achieve those expectations and goals in depressed states, which in turn may lead to negative self-appraisal and rumination. A final related hypothesis explaining the link between the tendency to ruminate and mania in bipolar disorder is rooted in the possibility that individuals with mania may have experienced more failures, as well as destructive complications, as a result of their mania (as opposed to failures due to their depression). According to this account [61], hypomania/mania may have led to more failures and in turn may lead to a greater likelihood to resort to self-blame, negative self-appraisal and rumination.

One problem with these three psychological accounts is the finding from Johnson and colleagues (2008) that patients with bipolar disorder engage in positive rumination (that is, positive self-appraisal) in response to positive affect in a hypomanic state. Similarly, Eisner and colleagues [62] found that there is a tendency to engage in positive generalization, similar to positive rumination, in response to successes. An account of why patients with bipolar disorder ruminate must take into account why they ruminate in response to both negative and positive affect.

## The tendency to ruminate in bipolar disorder and executive dysfunction

We propose that the link between the tendency to ruminate in manic and depressed states in bipolar disorder reflects executive dysfunction in bipolar disorder. Research on neuropsychological functioning in bipolar disorder reveals deficits in several cognitive domains, including attention, learning and memory, and executive functioning [63-66]. The executive dysfunction seen in bipolar disorder is present in euthymic, depressed and manic states, suggesting that the executive dysfunction is not state specific, but rather trait-like. In one study, investigating neuropsychological functioning in bipolar patients in manic, depressed and euthymic mood states, Martinez-Aran *et al*. [66] found that regardless of mood state, all patients with bipolar disorder showed impaired functioning on tests of executive functioning, including the Wisconsin Card Sort Task (WCST) and Stroop Color and Word Interference task. While a more recent study calls into question the presence of executive dysfunction in euthymic states in bipolar disorder, they nonetheless found a trend towards executive dysfunction in patients with bipolar disorder who are euthymic [67]. It is possible that the executive dysfunction present in patients with bipolar disorder in euthymic states is exacerbated in mania and depression.

In fact, in studies using tests of executive functioning, including attentional set-shifting [68,69], planning ability [70] and decision making [69,70], researchers consistently find deficits in executive functioning in mania. And the finding by Martinez-Aran *et al*. [66] of executive dysfunction in depressed states in bipolar disorder is consistent with the literature showing executive dysfunction in depressed states in MDD (see [71] for a review). Of note, many of the tasks used to assess executive functioning are tasks which incorporate multiple executive processes (for example, the WCST incorporates shifting attention as well as inhibition), so a fine grain approach to executive functioning as suggested by Miyake *et al*. [72] is not currently possible. However, such an approach would be invaluable at more precisely identifying particular executive deficits in bipolar disorder. This highlights the need for research into specific executive processes in bipolar disorder.

There is some initial evidence that the tendency to ruminate is associated with executive dysfunction. In a study by Davis and Nolen-Hoeksema [73], individuals with a tendency to ruminate were found to demonstrate perseverative tendencies and failures to maintain adaptive cognitive sets on the WCST. Similarly, Watkins and Brown [74] found that depressed patients who underwent a rumination induction and were asked to perform a random number generation task showed failures of inhibitory executive control compared to depressed participants who underwent distraction, and non-depressed participants. More recently, Whitmer and Banich [75] found that individuals with a tendency to ruminate have trouble inhibiting mental representations of previous task demands when they switch their attention to new task demands. Thus, rumination seems to be associated with executive dysfunction, particularly inhibitory executive control. Of course, given the dearth of studies, further research into executive functioning and rumination is much needed.

Taken together, the evidence for executive dysfunction in bipolar disorder and the relationship between rumination and executive dysfunction suggests that the tendency to ruminate may be symptomatic of executive dysfunction in bipolar disorder, specifically inhibitory executive dysfunction. Patients with bipolar disorder may experience an exacerbation in executive dysfunction when manic or depressed, which may lead to a tendency to ruminate because of a failure to inhibit self-focused thoughts of a positive or negative nature. This possibility invites investigation into the effects of mood states on executive functioning and rumination in bipolar disorder.

## Neural correlates of bipolar disorder and rumination

Research into neural correlates of bipolar disorder has increased dramatically in the last few decades with advances in structural and functional neuroimaging. The data for structural differences are somewhat mixed, with a few studies showing no differences between individuals with bipolar disorder and healthy controls in prefrontal regions [76-78] and amygdala [79], and other studies reporting differences in both prefrontal regions [80-83] as well as amygdala [84-87]. Two recent meta-analyses using region of interest analyses found no evidence of gray matter abnormalities [88,89], but more recently one meta-analysis study using voxel based morphometry (VBM), which uses a whole brain technique, found reductions in grey matter in the rostral anterior cingulate cortex and the right fronto insular cortex [90], while another meta-analysis study, also using VBM, found reductions in grey matter in the anterior cingulate and insula [91].

Functional neuroimaging studies comparing patients with bipolar disorder and healthy controls consistently report differences in prefrontal cortices, with early studies showing blood flow and/or metabolic abnormalities in overall frontal metabolism in patients with bipolar disorder compared to healthy subjects [92-96], and later studies suggesting more fine-tuned differences in activation in subgenual prefrontal cortex in patients with bipolar disorder compared to healthy controls [97].

More recently, studies using functional Magnetic Resonance Imaging (fMRI) while performing various tasks, such as presentation of emotional stimuli, the Stroop task and the Go/No-Go, and memory encoding tasks, consistently find differences in activation in prefrontal regions in patients with bipolar disorder across all mood states. Studies have found differences in activation in dorsolateral prefrontal cortex (DLPFC; BA9/46) [98,99], left ventral prefrontal cortex (VLPFC; BA47) [100], rostral ventral prefrontal cortex (VLPFC; BA10/47) [101], and orbital and medial prefrontal (BA 8/9) cortices in patients with bipolar disorder who are euthymic [100]. One study found differences in activation in left ventral prefrontal cortex (BA 10/47) in patients with bipolar disorder who were manic as well as those who were depressed [101]. Functional neuroimaging studies also consistently report differences in amygdalar activitation in response to negative stimuli relative to healthy controls in patients with bipolar disorder who are euthymic [98], depressed [102] and manic [103].

Additionally, there are two studies that suggest a link between activity in prefrontal cortices and amygdala in bipolar disorder. Yuerglen-Todd *et al*. [98] found that patients with bipolar disorder who are euthymic demonstrate a reduction in DLPFC (BA 9/46) activation and an increase in amygdalar activation in response to fear faces, relative to healthy controls. Ketter *et al*. [102] found that patients with bipolar disorder who were depressed had decreased global prefrontal and paralimbic cortical metabolism and increased metabolism in right amygdala, ventral striatum and thalamus. Taken together, these two studies suggest that decreased functional activation in prefrontal areas may reflect a loss of cognitive control or executive dysfunction, and a failure of emotion regulation, which may in turn lead to increased amygdala activation. Consistent with this, a recent meta-analysis of 65 fMRI studies in bipolar disorder by Chen and colleagues [104] found evidence for fronto-limbic activation in bipolar disorder, which was characterized by attenuated ventrolateral prefrontal cortex activation across emotional and cognitive tasks and enhanced limbic activation with emotional tasks.

In a recent review on the neurophysiology of emotion regulation, Phillips *et al*. [105] conceptualized a model of emotion regulation consisting of a voluntary emotion regulation system involving lateral prefrontal cortices (DLPFC and VLPFC) and an automatic emotion regulation system involving medial prefrontal cortices (OFC, subgenual ACC and medial dorsal PFC). The model is based on research in healthy controls which demonstrates increased activity in lateral prefrontal cortices in tasks of voluntary behavioral control to positive and negative stimuli (for example, suppressing facial reactions to emotional stimuli), voluntary attentional control (in which participants are directed to selectively attend to task-relevant emotional stimuli/inhibit distraction from task-irrelevant emotional stimuli, for example) and voluntary reappraisal (for example, decreasing negative affect from a negative stimulus); and increased activity in medial prefrontal cortices in tasks of automatic behavioral control (for example, decreased affect to emotional stimuli), automatic attentional control (implicit direction of attention to or away from emotional stimuli, for example, the Stroop task), and automatic reappraisal (for example, implicit appraisal of novel contexts).

Phillips *et al*. [105] show that there is an overlap with the above findings in bipolar disorder. They report mixed findings with regards to voluntary emotion regulation: studies of voluntary attentional control show reduced activation in lateral PFC and DLPFC regions, while studies of voluntary emotion regulation show greater activation in those same areas. The literature on automatic emotion regulation in patients with bipolar disorder is more consistent with studies of automatic attentional control showing reduced activitation in ventromedial PFC in bipolar disorder compared to healthy controls and studies of emotion regulation showing reduced activation within ventromedial PFC. Thus, overall, while it is clear that more research on emotion regulation in bipolar disorder is much needed, it is nonetheless apparent that there are differences in bipolar disorder in brain regions that are involved in emotion regulation.

Compared to the research on neural substrates of bipolar disorder, the research on neural substrates of rumination is relatively new. In fact, there have been less than a handful of studies, most of them in the last few years. These studies suggest overlap with brain regions implicated in affective dysregulation in bipolar disorder, including anterior cingulate, DLPFC, OFC, as well as amygdala, suggesting a common neural substrate for rumination and affective dysregulation in bipolar disorder. In one of the first studies, which investigated the relationship between the tendency to ruminate and amygdalar response to word stimuli, Siegle and colleages [106] found that in depressed individuals, the tendency to ruminate was moderately correlated with sustained amygdala activity following presentation of negative items.

Ray *et al*. [107] investigated the relationship between the tendency to ruminate and cognitive reappraisal after emotional responses in a non-clinical sample. They found that the tendency to ruminate was correlated with increases in amygdala response, when participants were instructed to increase negative affect, and with decreases in prefrontal regions, including anterior cingulate cortex and medial prefrontal cortex, when participants were instructed to decrease negative affect. More recently, Cooney *et al*. [108] looked at neural activity during a brief rumination induction, a concrete distraction induction, and an abstract distraction induction in depressed individuals and normal controls. They found that depressed individuals showed greater activation in the OFC (BA 11), subegenual cingulate (BA25), and DLPFC (BA 9) than normal controls during rumination compared to concrete distraction. Additionally, depressed participants showed greater activity than healthy controls in the rumination condition compared to abstract distraction in the amygdala, dorsal anterior cingulate (BA 24), rostral anterior cingulate (BA 32), DLPFC (BA 46), posterior cingulate (BA 31), and parahippocampus. More recently, Hamilton *et al*. found a correlation between the depressive rumination in depressed patients correlated with resting state activity in posterior cingulate and medial prefrontal cortices [109].

Notably, many of the brain regions implicated in bipolar disorder overlap with regions implicated in rumination, namely prefrontal cortical areas, including the anterior cingulate, DLPFC, and OFC, as well as the amygdala. The evidence for common neural substrates in bipolar disorder and rumination, particularly in prefrontal cortical regions, which are believed to be involved in executive functioning, is consistent with our hypothesis that executive dysfunction may underlie the tendency to ruminate in bipolar disorder. Patients with bipolar disorder may ruminate because they experience difficulty inhibiting their persistent self-focus, once it has been initiated. The fact that prefrontal brain regions implicated in bipolar disorder and rumination include regions involved in automatic emotion regulation (ACC, OFC) and voluntary emotion regulation (DLPFC) point to a common neural substrate for affective dysregulation as well. Thus, patients with bipolar disorder, due to differences in functioning in prefrontal cortical regions may experience difficulty inhibiting and regulating emotion, just as they have difficulty inhibiting their persistent self-focus in response to positive or negative affect (that is, rumination). In fact, rumination, more generally speaking, might be explained by differences in functioning in prefrontal cortical regions and a deficit in inhibiting self-focus. These hypotheses provide interesting lines of investigation that might be pursued in future neuroimaging studies.

## Conclusions and future directions

The mind in bipolar disorder, whether manic or depressed, is never quiet. Patients with bipolar disorder struggle and tend to ruminate in depressed states, just like their MDD counterparts. Unique to bipolar disorder is the finding that patients with bipolar disorder ruminate about positive things in hypomanic and possibly manic states, which raises several questions.

One question that arises is whether rumination contributes to worsening in hypomanic or manic states [50]. Research on rumination in depressed states [22] demonstrates that rumination maintains and even worsens depressed mood. However, it is unclear whether rumination plays a role in exacerbating or maintaining hypomanic or manic mood. On this point, Johnson *et al*. [50] not only found that patients with bipolar disorder ruminate in response to positive affect, but that it was explained by hypomanic symptoms. Also, Johnson and Tran [110] found that individuals with bipolar disorder show an increased focus on goals and increased confidence during manic states. Research is needed on the relationship between rumination in response to positive affect and future hypomanic or manic episodes.

Another question which arises is why do patients with bipolar disorder ruminate in response to positive affect? We know that in major depression, rumination begins with a desire to problem solve [28], but then evolves into a vicious cycle of repetitively focusing on one's symptoms of distress and how they came to be. One possibility is that rumination in response to positive affect is intended to maintain the positive mood. Johnson *et al*. [111] assessed the tendency to ruminate in response to positive affect using a measure which assesses the tendency to think about positive qualities of the self, positive affective experiences, and favorable life circumstances that might amplify the positive affect, and found that patients with bipolar disorder endorsed a greater tendency to ruminate to positive affect. It seems possible that such thinking might be geared towards maintaining a positive mood. However, research on the effects of positive rumination on mood is needed. Additionally, research on the reasons why people engage in positive rumination is also needed. It is also possible that patients with bipolar disorder ruminate in response to positive affect because the prospect of reward initiates a cycle of thinking which might maximize reward. Perhaps the positive affect serves as a sort of reward, and sets in cycle a desire for additional reward.

Finally, related to our hypothesis that the tendency to ruminate reflects executive dysfunction, there are several questions which warrant investigation. Is intact executive functioning protective? Are patients with bipolar disorder with little or no executive dysfunction less likely to ruminate in response to positive or negative affect? What are the neural mechanisms underlying the failure to inhibit rumination? It may be that the failure to inhibit rumination is simply a product of prefrontal dysfunction and an inability to inhibit certain cognitive processes as needed. Alternatively, it is possible that deficits in automatic and voluntary emotion regulation as posited by Phillips [105] perpetuates the cycle of rumination by affecting prefrontal functioning. Thus, there are several avenues of research related to rumination and bipolar disorder that have the potential to inform our understanding of what contributes to and maintains mood states in bipolar disorder.

## Abbreviations

ATQ: Automatic Thoughts Questionnaire; BDI: Beck Depression Inventory; DAS: Dysfunctional Attitudes Scale; DLPFC: dorsolateral prefrontal cortex; HDRS: Hamilton Depression Rating Scale; HPS: Eckblad and Chapman's Hypomanic Personality Scale; MDD: major depressive disorder; RSQ: Response Styles Questionnaire; SEQ: Rosenberg Self-Esteem Questionnaire; VBM: voxel based morphometry; VLPFC: left ventral prefrontal cortex; WCST: Wisconsin Card Sort Task

## Competing interests

SG and TD have no competing interests.

## Authors' contributions

SG and TD were involved in research for this manuscript and drafted the manuscript. Both authors read and approved the final manuscript.
